# Elderly Demand for Family-based Care and Support: Evidence from a Social Intervention Strategy

**DOI:** 10.5539/gjhs.v6n2p94

**Published:** 2013-12-06

**Authors:** Emmanuel Aboagye, Otuo Serebour Agyemang, Trond Tjerbo

**Affiliations:** 1 University of Oslo, Oslo, Norway; 2 Department of Economics and Management, University of Ferrara, Ferrara, Italy; 3 Department of Health Management and Health Economics, University of Oslo, Oslo, Norway

**Keywords:** elderly demand, family-based care and support, health insurance, social security, Ghana

## Abstract

This paper examines the influence of the national health insurance scheme on elderly demand for family-based care and support. It contributes to the growing concern on the rapid increase in the elderly population globally using micro-level social theory to examine the influence the health insurance has on elderly demand for family support. A qualitative case study approach is applied to construct a comprehensive and thick description of how the national health insurance scheme influences the elderly in their demand for family support. Through focused interviews and direct observation of six selected cases, in-depth information on primary carers, living arrangement and the interaction between the health insurance as structure and elders as agents are analyzed. The study highlights that the interaction between the elderly and the national health insurance scheme has produced a new stratum of relationship between the elderly and their primary carers. Consequently, this has created equilibrium between the elderly demand for support and support made available by their primary carers. As the demand of the elderly for support is declining, supply of support by family members for the elderly is also on the decline.

## 1. Background

### 1.1 Introduction to Problem

Ghana is predicted to witness a rise in the number of the elderly of its population. The percentage of the elderly of the country’s population is 6% (WHO, 2013), and it is estimated to be at least 8.4% by 2050 (UN, 2010). Currently, there is a decline in family arrangement of support for the elderly (Aboderin, 2004b; Ogwumike et al., 2005). And with this rapid increment in their (i.e the elderly) number, Ghana is bound to face certain challenges such as reduction in labour force, productivity losses, rising health care expenditure and so on. This concern has led to the development of welfare plans that place emphasis on certain social and economic securities that will afford elders the possibility to fully engage in society and become relatively independent (HelpAge International 2004; Ghana National Committee on Ageing, 2002).

The Social Security and National Insurance Trust (SSNIT), is one of such arrangements to protect people when they retire. However, the trust is not able to pay entitlements to beneficiaries on time when they retire, and do not accept contributions from informal sector workers where the largest share of the labour force can be found (Kaseke, 1999; Boon, 2007). This implies that many elders still remain poor and dependent when they can no longer work (Apt, 1996; Mba, 2007). Owing to the shortfalls of the SSNIT, the National Health Insurance Scheme (NHIS) was introduced in 2008 to promote health for the poor and the marginalised such as the elderly (NHIA, 2008). Per the definition of social security (Boon, 2007; Kaseke, 1999; Darkwa, 1997), the NHIS is a social arrangement, which has embedded characteristics to provide security and protection to the elderly. For instance, it helps the elderly to utilize health services virtually free.

A substantial number of studies have reported on social security system and the coping strategies of elders after retirement in relation to family support in Ghana (Kaseke, 1999; Boon, 2007; Apt, 1996). Apt (1996) in a study on coping with old age in a changing Africa mentioned the safety nets and coping strategies elders in Ghana adopt in their manifold living arrangements. In a study on gender disparities in living arrangements of the elderly in Ghana, Mba (2007) describes the types of living arrangements among the elderly. Also, a study by Kaseke (1999) on social security and elders in Ghana documents the extensive challenges of the social security among the poor and specifically, the elderly. The major features of the social security system have been studied and wider extension to more people has been recommended (Darkwa, 1997; Boon, 2007). Notwithstanding these, it is worth considering that these studies have not focused on how the NHIS affects the coping strategies and living arrangements of the elderly. This line of research, to the best of our knowledge, has largely been neglected in studies pertaining to social security among the elderly in Ghana. Therefore, this paper aims at filling this lacuna by examining the influence of the NHIS on elderly demand for family-based care and support. The questions posed are: 1) who are primary care-givers of the elderly? 2) What typical living arrangements do the elderly have? 3) How does the NHIS influence demand for family-based care and support for the elderly?

### 1.2 Health Care Financing and Health Insurance Scheme in Ghana

Currently, there is an on-going gradual shift from cash before service system of financing health care to insurance-based financing forms (Asante et al., 2008; Asenso-Okyere et al., 1998). The District Mutual Health Insurance Schemes (DMHIS), Private Commercial Health Insurance Schemes (PCHIS) and Private Mutual Health Insurance Schemes (PMHIS) are the scheme types found in Ghana (Atim et al., 2001; Government of Ghana, 2003). Private health insurance schemes are fewer and generally used by individuals and companies or groups to pay for medical cost of employees through premium deductions from salaries (Osei-Akoto, 2003).

The National Health Insurance Scheme (NHIS) is a merger of traditional social health insurance and mutual health insurance, which is administered centrally to pull together contributions (GOG, 2003). The NHIS is financed from taxes both direct and indirect into the National Health Insurance Fund (NHIF). SSNIT deductions, government budget allocations and annual premiums per insured persons also form part of contributions to the fund (NHIA, 2010). Contributions from the informal sector (i.e annual premiums) are based on income and ability to pay with no coinsurance, co-payment, or deductible required at the point-of-service. In addition, grants, donations, and gifts from donors support the funding of the NHIS (NHIA, 2010).

The NHIS was designed to promote social protection through risk equalization, cross subsidization, solidarity, as well as quality and equity in healthcare (NHIA, 2010; Nguyen et al., 2011). Individuals exempted from paying annual premiums are; children under 18 years, adults (70 years and above) and formal sector employees contributing to SSNIT (NHIA, 2008; MOH, 2009). General out-patient and in-patient care, oral health, eye care, comprehensive delivery care, diagnostic tests, generic medicines and emergency care are covered under the scheme (NHIA, 2008). However, specialized forms of care such as dialysis and organ transplants are not covered under the insurance. Services under government vertical programmes such as antiretroviral for HIV/AIDS treatment, drugs for immunization and family planning are not supplied under the NHIS.

The NHIS is criticised for not covering the extremely poor (Asante et al., 2008). Many families have less income and unable to pay annual contributions to benefit from health insurance (Arhinful, 2003; Jehu-Appiah, 2010). Notwithstanding this drawback, the scheme gives access to health care and replaces the ‘cash and carry’ system the country was witnessing before the implementation of the Scheme. It is contended that, the NHIS can help meet the rising health care cost of the elderly (Gobah et al., 2011; Jehu-Appiah et al., 2011). It is therefore, imperative to investigate the influence the NHIS can have on the elderly demand for family support.

### 1.3 Social Theories of Aging and Analytical Approach

Theories of aging can be used at the micro and macro-social levels of analysis to aid explanation (Bengtson et al., 2009; Pierce et al., 2010). However, some social processes in aging studies operate both within micro and macro levels (Bengston et al., 1997; Bengtson et al., 1999). From the macro-social level, modernisation theory is often used to explain the changes in family support for the elderly in both developed and developing economies (Apt, 1993; Gyekye, 1996; Aboderin, 2004a). According to this perspective, the collapse of extended family and shifts towards nuclear family explain the decline in family support for the elderly (Aboderin, 2004a). Contrariwise, the material constraints notion gives counter explanations to the family breakdown view (i.e modernisation theory). Currently, material constraints of families are the main cause for the decline of family support for the elderly in developing economies (HelpAge International, 2002; Apt, 1997).

However, both perspectives are limited in giving theoretical justification for the decline in family support for the aged. The modernisation perspective does not consider the role that material constraints play in the decline of family support. It focuses on changes in roles and norms, and the resulting decline of family support (Aboderin, 2004a). The material constraints viewpoint on the other hand, does not recognise the role values and norms play in the declining family support for the elderly (Aboderin, 2004a). Both perspectives do not marry well in explaining the inter-relationships between material and social changes. Moreover, both ideas are limited in connection to the influence a social intervention can have on demand for family support among the elderly. These limitations make the social constructionist perspective (which considers the interrelations between interventions and changes in behavior) an appropriate explanatory guide for this study.

### 1.4 Social Constructionist Perspective

Social constructionist perspective of aging reflects a long tradition of micro-level analysis focusing on individual social behaviour within larger structures of society. According to this idea, social change occurs when social structures and those that make up the society interact.

The theory in general explains what can be a multifaceted interrelationship between societal process at the micro level, the formation of public policy and the well-being of the elderly (Bengtson et al., 2009). It demonstrates how social processes can formulate old-age policy and how this policy shapes the experience of the aged. The interactions between social processes and old-age policy can have differential effects on differing subgroups within the aged population. And these interactions are interpreted within the social constructionist perspective. The effect of larger social forces (such as modernization and urbanization) on the elderly and how the elderly through exercising agency shape the social structures, which eventually shape their social context are all within the framework of social constructionist perspective.

There is a clear process of interdependence between the elderly and the social structures. Through this it can be understood that agency is embedded within social structures (Bengtson et al., 2009). This approach is useful and it is supported by other perspectives such as critical theories and the life course standpoint (Baars, 1991). However, this approach is criticised for making unclear macro-level effects- such as cohort, historical, and age stratification influences -when the individual is given much attention and less attention is given to the role social structures play.

In the context of this study, health insurance and the family can be explained as macro-social level structures. The family is described as a structure because it is a social security arrangement for people during old age. The elderly are the agents at the micro level of social interaction. By using the social constructionist theory, the attitudes of the elderly that will be observed in this study will be shaped by the health insurance intervention thus creating other influences on family support (through the interaction between the elderly and the intervention).

## 2. Method

Yin (2011) considers qualitative research approach as attractive- if not the mainstream- sort of research in both academic and professional operations. Since this research aimed at obtaining in-depth and extensive knowledge about primary care-givers of the elderly, their living arrangements and nature of their dependence on family support, qualitative research method was considered appropriate for the study’s analysis (Grbich, 1999; Yin 2009). In addition, in order for the study to obtain a comprehensive understanding about our informants in their natural setting, a case study design was employed to complement the qualitative research approach. This section lays out how the cases in the study were selected, followed by the data collection techniques, method of analysis, the study setting and the methodological challenges.

### 2.1 Case Selection

The procedure by which informants are selected from a population to become a sub-group of interest in a case study research is called case selection. Since the study wanted to discuss differing cases, the diverse case method which is a non-random purposive procedure was used to select our informants. This method has the capability to handle different cases within categories and also explain the outcome through these cases (Gerring 2008).

By the nature of our study and for the purpose of enhancing external validity, cases that reproduce characteristics of the population and also provide differences along the dimension of theoretical interest were selected. In all, six cases -aged 65 years and above- were selected. Eisenhardt (1989) argues that while more than 10 cases are not needed in a case study, less than 4 cases hardly provide the necessary and sufficient information. Therefore, the requirement for sufficiency of evidence was achieved in this research. In order to make sure that these selected cases were evenly distributed between the urban and rural areas of the region, 3 cases were selected from each area. This urban-rural mix setting used ensured wider discussion and comparable views instead of a one-sided view, if the study had chosen urban areas over rural areas or vice versa.

### 2.2 Data Collection Techniques

This study employed two data collection techniques: interviews and observation. Firstly, the study applied focused interviews with a semi-structured guide to gather information. This technique of interviewing was employed in that it has the ability to substantiate evidence that is perceived to be already established (Yin, 2009). The questions were in sections and had different, but related themes. The questions focused on primary care givers, living arrangements and self-support. Other questions on daily activity levels, health insurance coverage and family-based support were also asked to generate insight about elderly dependence. Personal information on informants such as age, gender, marital status, occupation, and residence were also asked. Secondly, in order to gather information that are sometimes difficult to get from interviews, observations are used as a substitute. Therefore, the study relied on direct observation to gather such information. Through this technique, the researchers were able to observe the social status of informants and the strength of connection between the elderly and the family or caretakers. The researchers were particularly keen on observing the activity level of the informants since chronological age alone cannot determine the daily activity elders can perform. Activity level in this study refers to how elders are able to do their everyday activities with or without any support.

The interviews were moderated by the researchers. The questioning and answering occurred in a calm and serene atmosphere that enabled the researchers to tape-record the responses with small tape recorder. Following Ravasi and Zattoni (2006), the various transcriptions were reinforced with interview notes and contact summary sheets. All interviews were carried in the Akan language, but during the report writing stage, all the quotations were translated into English. Informants were contacted to seek for their consent before they were interviewed. Similarly, informants were contacted if their direct quotes were used in the report writing in order to reduce misinterpretation of responses of the variables in the study (Crank et al., 2007). All names mentioned in the analysis are not original names of informants. The names are used to aid easy reading and understanding.

### 2.3 Analysis of Data

The cases selected were pattern matched using the cross-case technique. The technique combines two strategies in the analysis: 1) select interesting and diverse cases, which are then explained using the underlying theory of the study; and 2), address all rival cases (Yin 2009). It became relevant to use this technique since there were multiple cases in our analysis. Through cross-case synthesis, the transcribed texts were used to create a table (see table 1). This table shows data of individual cases under similar description. The selection of comments and observations from the data are brought under related broad themes in the table. The themes formulated from the research questions were systematically categorised and analysed. The variables examined were: gender, residence, living arrangement and so on (see table 1). For instance, living arrangements among elders were categorised as either living alone or with someone.

**Table 1:** Cross analysis of primary carers and support for the elderly

	Gender/Age	Primary caregiver	Living arrangement	Support from family
**Urban**				
Maa Adwoa	Female (71)	House help	Alone (with house-help)	None
Abra	Female (72)	Son	Alone (with house-help)	Pay rent
				Money for food
				Provide clothing
				Medical bills
				Other things
Wofa	Male (76)	Son	Alone	Other things
**Rural**				
Opanin Kofi	Male (85)	grandson	grandchildren	Provide food
				Provide clothing
				Medical bills
				Other things
Opanin Nkosuo	Male (83)	niece	Alone	Provide food
				Other things
Manuwaa	Female (80)	daughter	Daughter	Pay rent
				Provide food
				Provide clothing
				Medical bills
				Other things

Source: generated from the interviews by the authors for the purpose of the study

### 2.4 Study Area

The study was conducted in Ashanti region, which has the highest elderly population among all the regions in Ghana. The proportion of the population aged 65 years and above in the region increased from 2.5 per cent to 3.0 per cent in 1960 and 1970 respectively (GSS, 2009). By 1984, the elderly population in the region had grown to 3.6 per cent. The same age group had increased from 6.1 per cent in 2000 to 6.3% in 2010. The rationale that informed the selection of this region is twofold. First, the NHIS has a wider coverage in this region compared to other regions (NHIA, 2009). Second, respect towards the elderly and the feeling of belongingness to the family is held high in their cultural values (Van der Geest, 2002).

### 2.5 Challenges

Although the small sample size reduces the strength of generalisation, in order to make it possible, a replication method was applied in this study. Yin (2009) argues that replication logic is the same logic that underpins each case and be applied in all cases. The study applied similar approach for data gathering and this procedure was directed by a case study protocol. The case study protocol comprised the study’s overview, explanation of the purpose of the study and field procedures such as the application of the Activity of Daily Living (ADL) questions. This technique strengthens external validity of outcomes and makes the results closely representative of the entire population (Gerring, 2008; Yin, 2009).

## 3. Results

The family is essential in the Ghanaian culture because of weak social security institutions for the elderly (Nukunya, 1992; Gyekye, 1996). The composition of a family can vary and it can spread even beyond the traditional nuclear and extended families. In addition to procreation role the family plays in the society, it also gives financial assistance to family members and supports the elderly (Apt, 1993; Apt, 1996). This section of the paper identifies the individuals who are mentioned by the elderly as primary carers.

### 3.1 Primary Carers of the Elderly

Primary carers are those who support the daily activities of the elderly when they (i.e the elderly) increasingly become weak and have health challenges in doing many things on their own. Table 1 below highlights the cross analysis of primary carers and support the elderly receive from their family members. The daily life activities of the elderly were generated from the Activity of Daily Living (ADL) questions.

Abra is 72 years old, lives in an urban area and feels she can do everything. She has a son who is married and cannot accommodate Abra in his home, but he provides food, clothing, and pays medical bills and other necessities. Moreover, Abra depends on the son in regards to difficult daily activities. When Abra was asked about whom her primary carer was, this was what she had to say:

*‘‘I count on my son for help when the need arises. Sometimes when I have difficulty in breathing and my condition becomes very bad, he stays here or comes to me every day. I can also count on this little girl [‘house- help’] my son brought to stay with me. If the parents will agree to let me have her forever then she can also help. As the saying goes, do unto others what you want others to do unto you. I have taken good care of her and I expect that she reciprocates it in the same way if she wants to live with me. However if she decides to go, I cannot detain her. I will bless her before she leaves’’*.

71 year old Maa Adwoa in an urban area also had this say: *‘‘*[*A*]*t this age, I can cook, dress up, and bath. However, washing is difficult for me. It takes a lot of time to wash but I do it in bits until I finish. Carrying heavy things like water is impossible for me. I cannot do certain things alone, that is why I have this little girl with me. She helps me with certain things. For instance, she does errands since she cannot cook. Even though she stays with me, I still have to do a lot by myself. My situation is really sad since at my age I do not have anyone’’*.

From the above statement, Maa Adwoa feels she does not have anyone in times like these and this prompted the researchers to ask questions with respect to her family members. She answered that:

*‘‘I have a family, but it is God I count on whenever I need help. Family is nothing. My family members do not even recognise me as part of them and as a result, they do not bother themselves visiting me at all. If you have no children, then clearly you have no family. I live in this area with friends from my hometown. They care more about me than my own family. I depend on God all the time other than man. I live with this little girl* [‘*house-help’*] and *if, God forbid, she decides to leave one day, then that will be the beginning of my end. She is someone’s property but I take care of her. She in return serves me but if she decides to go I cannot say no’’*.

The bitter sounding Maa Adwoa feels her family has neglected her because she could hardly identify any member of the family who is willing to cater for her. Accordingly, she depends on her ‘house-help’ and close friends for support and care. The grocery store she operates is what provides her daily needs. However, she hopes that when she is too old, her family members will come to her aid since she has no children.

Manuwaa is 80 years old and lives in a rural area. Her health condition has made her weak. According to her, performing daily activities are extremely challenging. She cannot walk for long and has been advised to try to walk around all the time to keep fit. She lives with her daughter whom she identifies as her primary carer. Notwithstanding this, Manuwaa always feels she needs support and care from other family members. She has other children who help in providing her basic necessities such as rent, food, and clothing.

From the stories narrated and summarised in table 1 above, it is evident that the elderly still have ties with their family members in both urban and rural areas. However, this sort of ties is manifested between the elderly and their children who remain the primary carers. Usually support is tied with the needs and inabilities in the daily life of the elderly. Support with food, clothing, and payment of medical bills, as well as housing and psychological support are the common forms of assistance and care according to the elderly by their primary carers. Children who could not directly support and care for their elderly, usually offer monetary assistance to them (the elderly) on monthly basis or at irregular interval. In some cases, a ‘house-help’ or one of the family members takes the responsibility of care and support. These observations are consistent with the conclusions made by Aboderin (2004b) and Van der Geest (2002). Elderly women receive support from their family and in some instances, their children. However, the empirical fact reveals that, elderly women largely receive assistance from their children. This outcome is in line with Apt (1996) and Mba (2007) work on the primary care-givers of the elderly and the gender differences in support and care for the elderly respectively.

### 3.2 Living Arrangements of the Elderly

In times gone by, living arrangement that was common among the elderly was to live together with relatives. The relatives were mostly their children, grandchildren and others such as siblings, cousins and so on (Apt 1996). There is evidence that living arrangements of the elderly are gradually changing.

Manuwaa has 9 children but lives with one of them. According to her, her children are supportive but do not have time for her. With the exception of the one who lives with her, all her children are married. When she also gets married, Manuwaa may have to live alone. She feels this may happen very soon but she is certain that her children will continue to support her.

Both Opanin Nkosuo and Opanin Kofi are about the same age and reside in a rural area (see Table 1). They have lived and worked previously in so many places across the country. They decided to return to their maternal extended family to get assistance from family members in regards to some activities they cannot undertake. However, whilst Nkosuo still lives alone, Kofi lives with his grandchildren. The difference between them stems from their social status. On the one hand, Nkosuo still receives pension benefits from his former employers, which make him less dependent on family members. He has therefore, decided to live alone. It is his niece who only comes around to cook for him. On the other hand, Kofi lives with his family because of his financial status. With the exception of the national health insurance, Kofi does not receive any benefits from anywhere and therefore, has decided to live with his grandchildren.

The situation of living alone is entrenched among elderly men. Elderly women, who live alone more often than not, get support from their children. Moreover, it is more likely for elderly men with high social and economic protection to live alone. This result is consistent with previous studies done on living arrangements among the elderly in Ghana (Mba 2007; Van der Geest 2002).

### 3.3 Protection for the Elderly through Health Insurance

Informants responded to questions on how important health insurance is, as well as gave their personal views on health insurance as socio-economic security mechanism during old age. This discussion includes informants who apart from the health insurance coverage receive social security pension benefits.

The possession of health insurance is important for the study’s informants. Maa Adwoa mentions that things would have been difficult without health insurance ‘‘*because I am now able to go to hospital when I need health care. If someone wants to help you during sudden illness, they will ask whether you have health insurance. When people realize that you are insured, they are willing to take you to hospital. The taxi fare may not be a problem, but the medical bills can deter them from helping if you have no insurance. Before I was insured, I waited to get assistance before I showed up in hospital or clinic. Now things are not like that. You never know when you will get sick. It can happen anytime, so you should be prepared’’*.

Wofa is a retired accountant with 6 children but lives alone. His sister and one of his sons visit him frequently. Wofa always visits the regional hospital when he falls sick. He mentions that the kind of sickness he is suffering from makes the cost of medicine very high, but the health insurance covers some of his health care expenditure. He does not ask other family members for support. However, without the insurance he would have perhaps needed the family more or lived with someone. The social security and health insurance according to him, has provided protection and higher chances of living alone. He also believes *‘‘…it is a national commodity that everyone must have. Moreover, the mutual health insurance dwells on the number of people registered to operate, as they say: in numbers we have strength’’*. The health insurance coverage makes a difference for Wofa because with the pension benefits alone, he was finding it challenging to make ends meet.

Other informants mentioned some reasons that corroborate Wofa’s submission. In addition to safeguarding themselves from unexpected ailment and expenses, their grandchildren who are less than 18 years are also covered. This gives them a sense of responsibility to having contributed to their grandchildren’s health care cost.

The only informant who did not have health insurance recounted the importance health insurance and how laudable it is. Opanin Nkosuo is without health insurance but benefits from a pension plan that caters for his medical bills. Notwithstanding the benefits accruing from his pension plan, he still believes health insurance has somehow brought a certain level of economic freedom to some people. For this reason, he suggests that *“[i]t is important that everyone gets it because of the rising health care costs without health insurance”*. Because of the importance Opanin Nkosuo accords to the health insurance, by the time of the study’s data collection, he revealed to the researchers that he had started the registration process to get health insurance.

The health insurance is particularly important to the elderly in society. For some, it brings their families closer to them and also reduces the economic burden on family members who support them. It is therefore, imperative that the assessment of Boon (2007) should be given the necessary cognizance. The author contends that the way to improve and extend social security to the elderly is by ensuring that the traditional in-kind social security system arranged by the extended family go hand-in-hand with the formal social security structure- for instance the health insurance.

## 4. Discussion

The study has so far revealed that informants who enjoy pension benefits tend to depend less on family support as compared to those without such benefits. The question that can be asked is; if informants with such benefits are likely to depend less on family support, then can such evidence be established for informants who have health insurance? In other words, how does the health insurance influence the demand for family support by the elderly? This then leads this section of the study to find an answer to this question.

### 4.1 Demand for Family Support

Health insurance gives the elderly enough treatment and medicine without any cost. Although the attitude of the elderly towards health service utilization has not drastically changed, health care has become more accessible with health insurance. With the health insurance, elders now decide on their own, and do not have to wait for the ‘‘green light’’ from their primary carers. This implies that with the health insurance intervention, the elderly have changed from the “wait-and-see behaviour” to “check-now attitude”.

One of the informants, Wofa, believes his ill-health would have made his demand for family support higher if the cash-down before service system was still in place. He explains that, the social security pension benefit is not all that much and normally delays in reaching him on time thus making him depend on the health insurance for his health care needs. He further says that, the health insurance has lessened his dependence on his family.

The change in behaviour on demand for family support is evident from the informants’ comments. Reponses such as: ‘‘*I feel less of a burden for them’’*; ‘‘*I don’t ask them for support*’’; and ‘‘*I don’t bother them these days so much*’’ all point to the fact that gradually, the elderly are reducing their dependence on family support. The word, ‘them’ refers to primary carers mostly children or other family members.

Even though the breakdown of family and changes in family size can also explain this phenomenon (ie. the less dependence of elderly on their families), the elderly still have ties with their families and somehow get support as evidenced in the study’s analysis. Therefore, interactions that influence the decline of elderly dependence on family support may be outside the modernisation theory. It is also true that families are now using their limited resources to support the younger generation other than the elderly. Therefore, as active agents with fewer resources, who receive less material support from their families, they are expected to look elsewhere for support and protection by making use of available interventions that are aimed at reducing their demand for family support. Somehow, this outcome is logical since the elderly who are already not getting enough from their families will tend to depend on other social interventions thus reducing their dependence on their families. It is this interaction between the social intervention and the elderly which influences the declining dependence of the elderly on family support. The health insurance has removed health expenditure from the support list, which form part of the demand of the elderly from their children.

### 4.2 Differences in Elderly Dependence on Family Support

The faces of dependence include; life-cycle, physical, psychological, and economic and financial dependence (Baltes, 1996; Fine et al., 2005). All these types of dependence tend to occur in old age and are influenced by several factors including social structures such as policies on aging, elderly care interventions and so on. In this study, dependence is found to be different within and between age groups.

The empirical facts highlight that the elderly, who are between 65 and 75 years old and still active tend to be less financially dependent on their families. This may be so because of the reduction in their spending on medical costs. Also, within this same age group, the elderly with ill-health and are inactive are inclined to depend on their families.

Furthermore, the findings reveal that the elderly who are 75 years and above have the tendency to be financially and physically dependent on their families. In this age group, they need physical support with daily activities almost every day. Moreover, within this same age group, elders have information on physical activities from the health centres, which make them less dependent on physical care and support from their families. This implies that, elderly care has partially shifted from families to health care centres.

In conclusion, dependence on family support among the elderly may not decline in all the faces of dependence, as mentioned earlier. However, there are declines in financial, economic and in some cases, physical dependence on the family, mainly as a result of the health insurance intervention. This is consistent with the findings of Kinsella et al. (2005) that the elderly who have formal social security and health insurance are more independent of their families.

### 4.3 What Happens to Family Support?

This section of the study addresses the interaction between the elderly and the health insurance, and how this interaction influences the kind of support the elderly receive from their families.

The observable facts of the study highlight that the children of the elderly remain the primary source of support for them. However, the interaction between the elderly and the health insurance intervention has created a new form of relationship between the elderly and their primary support givers (ie. their children). Accordingly, this has produced a balance between demand made by the elderly and support given by their children. Thus as the demand of the elderly for support is declining, supply of support by family members for the elderly is also on a decline. The current declining demand of the elderly for family support also says a lot about the importance of the social intervention. This intervention has made the elderly less demanding in regards to healthcare costs and other necessities.

The findings also reveal that some of the elderly live alone, but it cannot conclusively be established that the health insurance alone could have altered their living arrangements. Other factors such as the social status of the elderly and changes in the family set-up also influence the living arrangements of the elderly. Meanwhile, the change in the family set-up is the reason the elderly depend less on family resources when they are exposed to other beneficial social interventions such as the health insurance.

The difference in age and levels of activity of elders are critical determinants of family support. In this study, it has been shown that some of the elderly needed the support and care from their families because of their health state and their incapableness. The reason is that, it may not be every elderly person that can be completely independent of his/her family (Kittay, 1999; Fine et al., 2005). This means the family is still needed since health insurance alone cannot lead to the realization of the decline of all the faces of dependence. Therefore, the importance of the health insurance cannot be overstated in such a way that family support and care are relegated to the background. In a nutshell, both the health insurance and the family can work hand-in-hand in providing health assistance to the elderly.

## 5. Conclusion

The study examines the influence of the national health insurance scheme in Ghana on elderly demand for family-based care and support. It concludes that health insurance is an important social intervention at old age. This stems from the benefits-such as economic freedom, precautionary measure, and easy healthcare accessibility- the elderly derive from this social intervention.

It has also been revealed that the elderly still have social connections with their family members in both urban and rural areas. These kinds of social connection are noticeable between the elderly and their children, who remain the primary carers. Furthermore, aside the NHIS, there are other factors (such as social status of the elderly and changes in family set-up) that influence the living arrangements of the elderly.

The interaction between the elderly and the NHIS has produced a new stratum of relationship between the elderly and their primary carers. Consequently, this has created equilibrium between elderly demands for assistance made available by their primary carers. As the demand of the elderly for support is declining, supply of support by family members for the elderly is also on the decline.

The study therefore, recommends that since social interventions complement family support to make the elderly more independent of their families, other well-structured social security interventions may be required in the future to make the elderly more independent. Also, since social interventions alone cannot ensure total independence, the importance of family system should not be underestimated.

This is a study to examine the influence of the national health insurance scheme on elderly demand for family-based care and support in Ghana. Therefore, the findings cannot be applied to other developing economies. Also, the sample does not represent the entire elderly population of Ghana. Accordingly, the observable facts cannot be generalized to cover other elders who have not been included in this research. However, the study’s results can be applied to elderly population in the country in an analytical sense. By applying an inductive reasoning, the findings of the study can be applied to provide relevant understanding on how health insurance can influence the demand for family-based care and support.
